# Inhibition of *Staphylococcus aureus* cocktail using the synergies of oregano and rosemary essential oils or carvacrol and 1,8-cineole

**DOI:** 10.3389/fmicb.2015.01223

**Published:** 2015-11-03

**Authors:** Vanessa G. Honório, Jéssica Bezerra, Geany T. Souza, Rayssa J. Carvalho, Nelson J. Gomes-Neto, Regina C. B. Q. Figueiredo, Janaína V. Melo, Evandro L. Souza, Marciane Magnani

**Affiliations:** ^1^Laboratory of Microbial Processes in Foods, Department of Food Engineering, Technology Center, Federal University of ParaíbaJoão Pessoa, Brazil; ^2^Laboratory of Food Microbiology, Department of Nutrition, Health Sciences Center, Federal University of ParaíbaJoão Pessoa, Brazil; ^3^Department of Microbiology, Centro de Pesquisas Aggeu Magalhães–FIOCRUZ/PE, Federal University of PernambucoRecife, Brazil; ^4^Laboratory of Electron Microscopy, Centro de Tecnologias Estratégicas do NordesteRecife, Brazil

**Keywords:** *S. aureus*, food matrices, rosemary, oregano, essential oil, antimicrobial activity

## Abstract

This study assessed the inhibitory effects of the essential oils (EOs) from *Origanum vulgare* L. (OVEO) and *Rosmarinus officinalis* L. (ROEO), as well as of the its majority individual constituents (ICs) carvacrol (CAR) and 1,8-cineole (CIN), respectively, combined at subinhibitory concentrations against a cocktail of *Staphylococcus aureus*. The Minimum inhibitory Concentration (MIC) of both OVEO and CAR against *S. aureus* cocktail was 1.25 μL/mL, while for ROEO and CIN the MIC value was 10 μL/mL. The Fractional Inhibitory Concentration Index of the combined EOs or ICs was ≤0.5 indicating a synergic interaction. The incorporation of OVEO and ROEO or CAR and CIN at different combinations in cheese and meat broths caused a decrease (*p* ≤ 0.05) of initial counts of *S. aureus*. Combined application of 1/8 MIC OVEO and 1/4 MIC ROEO or 1/4 MIC CAR and 1/4 MIC CIN in meat and cheese samples reduced (*p* ≤ 0.05) the viable cells counts and caused morphological changes in *S. aureus* cells, such as cell shrinkage and appearance of blebbing-like structures on cell surfaces. However, in cheese and meat samples the decrease in viable cell counts was smaller (*p* ≤ 0.05) than that observed in cheese and meat broths. These findings reinforce the potential of the use of OVEO and ROEO or CAR and CIN in combination to control *S. aureus* in cheese and meat matrices.

## Introduction

*Staphylococcus aureus* is a pathogen frequently associated with food outbreaks causing a typical intoxication through the ingestion of enterotoxins pre-formed in foods (Zeleny et al., 2015). Cheeses and meats have been frequently involved in major outbreaks of staphylococcal intoxication worldwide (Wang et al., 2013; Zeleny et al., 2015). Thus, the development of strategies to control this bacterium in these food matrices is of great importance for researchers and food industry (Pimentel-Filho et al., 2014; Pesavento et al., 2015).

From the middle of the last century, synthetic compounds have been used as additives in foods to control the growth and survival of pathogens, such as *S. aureus*, and give them a long shelf life and safety (Pesavento et al., 2015). The increased concern about the toxic effects of synthetic compounds used to preserve food matrices, as well as, the growing resistance of foodborne pathogens to these classic preserving agents increased the interest in natural antimicrobial substances, such as plant essential oils (EOs; Carvalho et al., 2015; Oliveira et al., 2015). Among the EOs that are generally recognized as safe (GRAS) in doses usually used in food and drinks (Food and Drug Administration [FDA], 2009) the EOs from *Origanum vulgare* L. (oregano – OVEO) and *Rosmarinus officinalis* L. (rosemary – ROEO) have showed promising results to control *S. aureus* in cheese (Asensio et al., 2015) and meat (Barros et al., 2009; Pesavento et al., 2015). Although many individual constituents are present in OVEO and ROEO, the activity of these EOs against *S. aureus* has been related to their majority constituents (ICs) carvacrol (CAR) and 1,8-cineole (CIN), respectively (Azerêdo et al., 2012; Oliveira et al., 2015).

Some studies have reported that the required amounts of EOs (and their ICs) needed to establish the desired antimicrobial effects in foods could exceed the threshold of food acceptability considering the taste and odor, which are frequently described as unpleasant to consumers (Gutierrez et al., 2008; Klein et al., 2013). Thus, the combined use of EOs or their ICs at subinhibitory concentrations exploiting the synergistic interactions may provide the balance between sensory acceptability and antimicrobial efficacy (Azerêdo et al., 2011; Oliveira et al., 2015).

The available reports of the efficacy of OVEO and ROEO and their ICs against *S. aureus* were in general obtained with strains in pure culture that do no reproduce the natural occurrence of microorganisms in food matrices (Romero et al., 2010). Still, some authors have shown that higher concentrations of EOs are required to establish the bactericidal effects in foods than in synthetic media (Burt, 2004; Gutierrez et al., 2008).

The concept of cocktail was introduced for physiological studies on foodborne pathogenic bacteria, primarily in acquisition of data as a way of determining the extreme of growth limits for particular species (Buchanan et al., 1993). It has been suggested that the use of bacterial strain cocktails could minimize the expected variation existent between different isolates of the same species, as well as the influence of the strain, obtaining an average tendency of strains behavior within species (Gibson et al., 1987; Romero et al., 2010). According to the conclusion of Chen et al. (2013), when a bacterial cocktail is constructed for experiments associated with food safety, strains with robust resistance behavior should be included. However, studies about the antimicrobial effects of the combined application of OVEO and ROEO, or CAR and CIN against *S. aureus* cocktails cultivated in food matrices, are still scarce.

Considering these aspects, this study evaluated the inhibitory effects of the combined application of OVEO and ROEO, and the majority ICs found therein CAR and CIN, respectively, against a cocktail composed by different *S. aureus* strains, as assessed by minimum inhibitory determination, fractional inhibitory concentration index and decrease of viable cell counts in cheese and meat-based broth, as well as in cheese and meat samples over time. Still, the effects of the tested EOs or ICs combinations on the bacterial cell surface in meat samples were observed.

## Materials and Methods

### EOs and ICs

The essential oils from *O. vulgare* L. (batch SZB1206; density at 20°C: 0.90; refractive index at 20°C: 1.47), and ROEO (batch ROSTUN04; density at 20°C: 0.94; refractive index at 20°C: 1.51) obtained by steam distillation, were purchased from Laszlo Ind. Com. Ltd. (Minas Gerais, Brazil). The CAR and CIN were purchased from Sigma Aldrich (Sigma, France). Emulsions of the essential oils or constituents were prepared in brain heart infusion broth (BHI, Himedia, India) with a range of concentrations (80–0.312 μL/mL) using Tween 80 (1%, v/v; Sigma–Aldrich, USA) as an emulsifier (Monte et al., 2015). Tween 80 at the higher assayed concentration (1%, v/v) presented no inhibitory effects against the assayed *S. aureus* cocktail.

### Test Strains and Growth Conditions

A panel of five *S. aureus* strains (LPM 5, LPM 27, LPM 33, LPM 52, and LPM 84) isolated from food-contact surfaces of food services in Hospitals of the city of João Pessoa (Paraíba, Brazil) was used as test organisms. The strains were selected from 100 *S. aureus* strains previously isolated, considering their capability to produce biofilm (stronger biofilm producers) and low similarity in molecular genotyping using RW3A rep-PCR (Liao et al., 2006; Rodrigues et al., 2015). During the investigation, the stock cultures were maintained in cryovials at –80°C.

The *S. aureus* cocktail was obtained by preparing suspensions in sterile saline solution (0.85% NaCl, p/v) from overnight cultures grown in BHI agar at 37°C. Each strain was grown in BHI broth at 37°C for 18–20 h (until late exponential growth phase), harvested by centrifugation (4500 *g*, 15 min, 4°C), washed twice in sterile phosphate-buffered saline (PBS) and re-suspended to obtain standard cell suspensions with OD measurements of 0.1 at 625 nm (OD_625_) to provide viable cell counts of approximately 10^8^ counting forming units per milliliters – CFU/mL (McMahon et al., 2008). The *S. aureus* cocktail was obtained using a ratio of 1:1:1:1:1 of each strain.

### Identification of OVEO and ROEO Constituents

The chemical composition of OVEO and ROEO was analyzed using a gas chromatography–mass spectrometry GCMS-QP2010 Ultra Shimadzu (Tokyo, Japan) with a capillary RTX-5MS column (5% diphenyl/95% dimethyl polysiloxane; 30 m × 0.25 mm × 0.25 μm). Helium (high purity) was used as a carrier gas at a flow rate of 0.99 mL/min. The oven temperature was increased from 60°C to 240°C at a rate of 3°C/min. The injector and detector temperatures were maintained at 250°C. The injection volume was 1 μL for OVEO or ROEO diluted with hexane (1:10). The mass spectrometer was operated in electron impact mode with a source temperature of 200°C, an ionization energy of 70 V, and a scan variation of 40–500 m/z scans at 3.33/s. Identification of the individual components was based on a comparison with the mass spectra of library of GC/MS, NIST/EPA/NIH Mass Spectral Database (Version 1.7). After normalizing the areas of each detected constituent, the results were expressed as a percentage area (%; Carvalho et al., 2015).

### Determination of the Minimum Inhibitory Concentration (MIC)

The MICs against *S. aureus* cocktail inocula were determined according to procedures described by Clinical and Laboratory Standard Institute [CLSI] with minor modifications related with the use of a stain to verify the (no) inhibition of bacterial growth (Wiegand et al., 2008; CLSI, 2012). Ninety-six-well plates were prepared by dispensing 50 μL each of OVEO, ROEO, CAR, or CIN solutions. Then, 50 μL of bacterial suspension (6 log CFU/mL) were added to each well, providing final concentration of 40–0.312 μL/mL. The microplate was wrapped loosely with cling wrap to prevent bacterial dehydration and ensure that the antimicrobials compounds would not volatilize. Each plate included controls without EOs or ICs. The systems were incubated at 37°C for 24 h and after the incubation period, a 30 μL aliquot of resazurin (0.01 g/100 L, w/v; Inlab, Brazil) prepared in aqueous solution was added to each well. Color changes were assessed visually after 20 min of incubation at 37°C. Bacterial growth was indicated by a color change in each well from purple to pink (or colorless). The MIC value was confirmed as the lowest concentration capable of inhibiting the growth of the strains.

### Determination of Fractional Inhibitory Concentration Index (FICI)

The checkerboard method was performed using broth (BHI) microdilution tests to obtain the FICI for the combined application of OVEO and ROEO or CAR and CIN against the *S. aureus* in cocktail. The FICI was calculated using the following formula:

$$\frac{MIC\,of\,\,compound\,A\,in\,\,combination}{MIC\,of\,compound\,B\,alone}\,+\frac{MIC\,of\,\,compound\,B\,in\,\,combination}{MIC\,of\,compound\,B\,alone}$$

OVEO and ROEO or CAR and CIN were assayed alone at MIC and at combinations of subinhibitory concentrations (1/2 MIC, 1/4 MIC, 1/8 MIC, 1/16 MIC, and 1/32 MIC). The results were interpreted as synergy (FICI ≤ 0.5), addition (0.5 ≤ FICI ≤ 1), indifference (1 ≤ FICI ≤ 4), or antagonism (FICI ≤ 4) (Gutierrez et al., 2008; Iten et al., 2009).

### Preparation of Cheese and Meat Broth

The effects of combined OVEO and ROEO or combined CAR and CIN on bacterial cell counts were studied in cheese and meat broths and in cheese and meat samples to simulate environmental conditions found by *S. aureus* in foods. The cheese [semi-hard cheese (*coalho*)] and meat (ground beef) samples used for preparing the broths and in assays to verify the bacterial cell counts were purchased from a local retail supermarket in João Pessoa (Brazil). Before the assays, the hygienic-sanitary conditions of the cheese and meat samples were assessed according to current sanitary standards established by the Brazilian legislation (Brazil, 2001), which establishes the limits of total and thermotolerant coliforms (45°C), coagulase-positive *Staphylococcu*s (*S. aureus*), *Salmonella* sp., and *Listeria monocytogenes*. Microbiological analyses were performed according to standard methods described elsewhere (American Public Health Association [APHA], 2001), and the obtained data demonstrated the satisfactory sanitary quality of the cheese and meat samples, including the low counts of *S. aureus* (≤2 log of counting forming units per gram – CFU/g).

To obtain cheese broth, the cheese (160 g) was cut into pieces of uniform sizes (1 cm × 1 cm × 1 cm), added to 1000 mL of sterile distilled water and hand-mixed with a sterile glass stem for 5 min to ensure even homogenization. Subsequently, the mixture was placed in a thermostatic water bath (42°C, for 50 min) and vacuum-filtered using Whatman n° 1 filter. The filtrate was sterilized in an autoclave for 15 min (1.21 atm; Neviani et al., 2009). For preparing meat-based broth, 500 g of beef lean meat type, trimmed of fat and whole external pieces of uniform size (3 cm × 3 cm × 3 cm) were boiled (90°C) in 1000 mL of distilled water for 30 min. Then, the liquid portion of the cooking material was separated from the solid material, vacuum filtered using Whatman n° 1 filters and the filtrate was sterilized using autoclave for 15 min (1.21 atm). Broths were stored at –20°C in aliquots of 50 mL, and when required one aliquot was thawed under refrigeration (7 ± 1°C) and used for the assays.

The physicochemical characterization (moisture, ash, fat, protein, and carbohydrate amounts) of cheese and meat broths was performed according to standard procedures (Association of Official Analytical Chemist [AOAC], 2012). The values of the assessed parameters were: moisture, 98.4 g/100 g; ash, 0.37 g/100 g; fat, 0.39 g/100 g; protein, 0.7 g/100 g; and carbohydrates, 0.23 g/100 g for the cheese broth; and moisture, 97.75 g/100 g; ash, 0.21 g/100 g; fat, 0.43 g/100 g; protein, 0.96 g/100 g; and carbohydrates, 0.65 g/100 g for the meat broth.

### Effects on Viable Cell Counts in Cheese and Meat Broth

The assays to verify the effects on the viable cell counts of *S. aureus* in cheese and meat broth over 24 h were carried out with the combinations of EOs or ICs at subinhibitory concentrations that inhibited the bacterial growth in assays of FICI determination. Combinations of OVEO and ROEO assayed were 1/4MIC + 1/8MIC; 1/4MIC + 1/4MIC; and 1/8MIC + 1/4MIC, respectively. The constituents CAR and CIN were tested at combinations 1/4MIC + 1/2MIC; 1/2MIC + 1/2MIC; and 1/4MIC + 1/4MIC, respectively. Initially, 150 μL of the cocktail suspension (∼8 log CFU/mL) were inoculated in 2850 μL of separated cheese broth or meat broth samples containing the EOs or ICs combined at the desired final concentration. The different systems (final viable cell counts of ∼6 log CFU/mL) were gently hand-shaken for 30 s, and then incubated at 7°C for 24 h. At intervals of 0 (immediately after homogenization of the system), 1, 2, 4, 8, 12, and 24 h of post-incubation (exposure time), an aliquot of 100 μL of each system was serially diluted in sterile saline solution, and then 20 μL of each dilution were dispensed in Baird–Parker agar + egg yolk solution and potassium tellurite (Himedia, India) using the microdrop inoculation technique (Herigstad et al., 2011). Control systems without the addition of the combined tested EOs or ICs were assayed similarly. The plates were incubated at 37°C for 24–48 h. Plates inoculated with aliquots collected from cheese or meat broths containing EOs or ICs were always incubated for 24 h longer than those obtained from control assays. The results were expressed as log CFU/mL.

### Effects on Viable Cell Counts in Cheese and Meat

The assays to verify the effects of combined EOs or ICs on viable cell counts of *S. aureus* cocktail in cheese and meat samples over 72 h were carried out with combinations of 1/8MIC OVEO + 1/4MIC ROEO and 1/4MIC CAR + 1/4MIC CIN, considering the bactericidal effects noted in assays using cheese and meat broths. For this, in each of four different systems (each referring to a specific cold storage period, as follow 0 h: first system – just after homogenization of the system, 24 h: second system, 48 h: third system and 72 h: fourth system) containing 20 g of macerated cheese or ground beef (taken from the same macerate sample pool), 10 mL of sterile saline solution, 1 mL of the tested bacterial suspension (∼8 log CFU/mL) and the desired final concentration of EOs or ICs were added. The systems were mixed using a sterile glass stem for 5 min to ensure even homogenization (final viable cell counts of ∼6 log CFU/mL) and incubated at 10°C. At each pre-established exposure time, 70 mL of sterile saline solution were added to the corresponding system, homogenized and serially diluted (10^-1^–10^-5^) in sterile saline solution (Carvalho et al., 2015). Subsequently, 20 μL aliquots of each dilution were dispensed in Baird–Parker agar + egg yolk solution and potassium tellurite (Himedia, India) to count viable cells using the microdrop inoculation technique (Herigstad et al., 2011). Control systems without the addition of combined EOs or ICs were assayed similarly. The plates were incubated at 37°C for 24–48 h. Plates inoculated with aliquots collected from cheese or meat containing EOs or ICs were always incubated for 24 h longer than those obtained from control assays. The results were expressed as log CFU/mL.

### Scanning Electron Microscopy (SEM) Analysis

Samples (10 g) of ground beef containing or not the combined EOs or ICs (1/8MIC OVEO + 1/4MIC ROEO or 1/4MIC CAR + 1/4MIC CIN) were inoculated with the *S. aureus* cocktail (∼8 log CFU/mL) and mixed with a sterile glass stem for 5 min to ensure even homogenization (final viable cell counts of ∼6 log CFU/mL). Then the systems were incubated at 7°C during 24 h. After this time, samples were fixed with a 2.5% (w/v) glutaraldehyde 4% paraformaldehyde solution in 0.1 M cacodylate buffer (pH 7.2) for overnight and post-fixed in 1% Osmium tetroxide solution diluted in Cacodylate buffer 0.1 M for 1 h at room temperature. Fragments of meat or cheese were then taken and dehydrated in an ethanol, dried in a critical point dryer (Bal-Tec, USA) and mounted in a stub and metalized with gold (20 nm thickness) using sputter coater (Balzers, USA; Oliveira et al., 2015). The samples were examined on a Quanta 200 FEG scanning electron microscope (FEI Co., USA) at an accelerating voltage of 30 kV.

### Statistical Analysis

All assays were performed in triplicate in three independent experiments, and the results were expressed as an average of the assays. For MIC determination assays, the results are expressed as modal values because values were the same in all repetitions. For viable cell counts assays, analysis was performed to determine differences (*p* < 0.05) in counts using ANOVA, followed by *post hoc* Tukey’s test. Sigma Stat 3.5 computer software (Jandel Scientific Software, San Jose, CA, USA) was used for the statistical analysis of the data.

## Results

### Identification of OVEO and ROEO Constituents

Analysis of constituents of OVEO and ROEO identified 16 and 13 constituents, respectively (**Table 1**). The constituents detected at the highest amounts in OVEO were carvacrol (66.9%), *p*-cymene (12.9%) and γ-terpinene (7.7%). Other compounds, such as myrcene (1.6%), linalool (1.5%) and α-pinene (1.8%) were detected in minor amounts. For ROEO, cineol (33.4%), camphor (14.2%) and α-pinene (13.8%) were detected in higher amounts. Other compounds, such as *p*-cymene and borneol, were detected in a range of 3.2–1.9%.

**Table 1:** Constituents identified in the essential oils from *Origanum vulgare* L. (OVEO) and *Rosmarinus officinalis* L. (ROEO).

Constituents ^∗^	Area (%)	
	
	OVEO	ROEO
α-thujene	0.5	–
α-pinene	1.8	13.8
Camphene	0.7	8.2
ß-pinene	0.6	7.0
Myrcene	1.6	1.8
δ-3-careen	0.1	–
α-terpinene	1.7	1.1
ρ-cymene	12.9	3.2
Limonene	0.3	5.6
1,8-cineole	0.6	33.4
*Cis*-ocimene	0.3	–
*Trans*-ocimene	0.3	–
Linalool	1.5	–
Camphor	–	14.2
Borneol	–	1.9
γ-terpinene	7.7	–
Verbenone	–	2.3
Bornyl-acetate	–	1.1
Carvacrol	66.9	–
ß-caryophyllene	1.6	2.3


*^∗^Constituents identified in each essential oil are expressed as the percentage area (%). (–), not detected.*

### MIC and FICI Assays

The MIC of both OVEO and CAR against *S. aureus* cocktail was 1.25 μL/mL, while for both ROEO and CIN, the MIC value was 10 μL/mL (threefold higher).

The results of the FICI assay showed a synergistic interaction for the combined application of OVEO and ROEO or CAR and CIN (**Table 2**). The FICI of the combined EOs was 0.375 and of the combined ICs was 0.5. Test strains cocktail displayed capability to grow at the tested subinhibitory concentrations of the EOs or ICs when applied alone (data not showed).

**Table 2:** Combined subinhibitory concentrations assayed on the checkerboard assay: concentration ratios [μg/mL] of the essential oils from *O. vulgare* L. (OVEO) and *R. officinalis* L. (ROEO) (OVEO/ROEO) or carvacrol (CAR) and 1,8-cineole (CIN), and Fractional Inhibitory Concentration Index (FICI) values against *S. aureus.*

OVEO/ROEO	[OVEO]	[ROEO]	FICI
1/4MIC + 1/8MIC	0.31	1.25	0.375
1/4MIC OVEO + 1/4MIC ROEO	0.31	2.5	0.5
1/8MIC OVEO + 1/4MIC ROEO	0.15	2.5	0.375

**CAR/CIN**	**[CAR]**	**[CIN]**	**FICI**

1/4MIC + 1/2MIC	0.31	5	0.75
1/2MIC + 1/2MIC	0.62	5	1.0
1/4MIC + 1/4MIC	0.31	2.5	0.5


### Effects on Bacterial Viable Cell Counts in Cheese and Meat Broths

**FIGURE 1:**
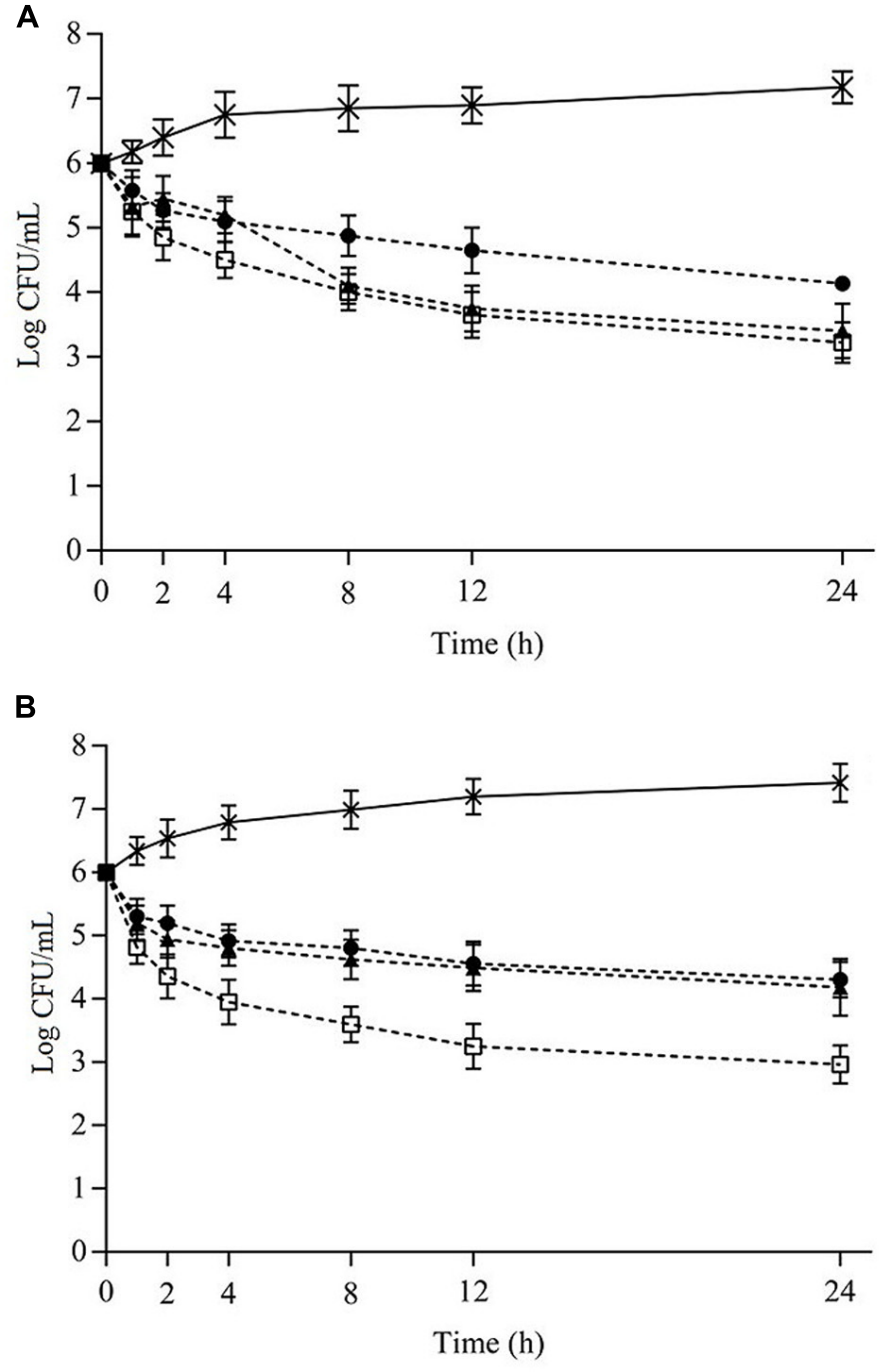
**Viable cell counts (Log CFU/mL) of *Staphylococcus aureus* cocktail in cheese broth (7°C) as a function of different combinations of the essential oils from *Origanum vulgare* L. (OVEO) and *Rosmarinus officinalis* L. (ROEO) **(A)** or carvacrol (CAR) and 1,8-cineole (CIN) **(B)**.** **(A)**: (•) 1/8 MIC OVEO + 1/4 MIC ROEO; (□) 1/4 MIC OVEO + 1/8 MIC ROEO; (▲) 1/4 MIC OVEO + 1/4 MIC ROEO; **(B)**: (•) 1/4 MIC CAR + 1/4 MIC CIN; (□) 1/2 MIC CAR + 1/4 MIC CIN; (▲) 1/2 MIC CAR + 1/2 MIC CIN. (^∗^) Control system (without OVEO and ROEO or CAR and CIN).

**FIGURE 2:**
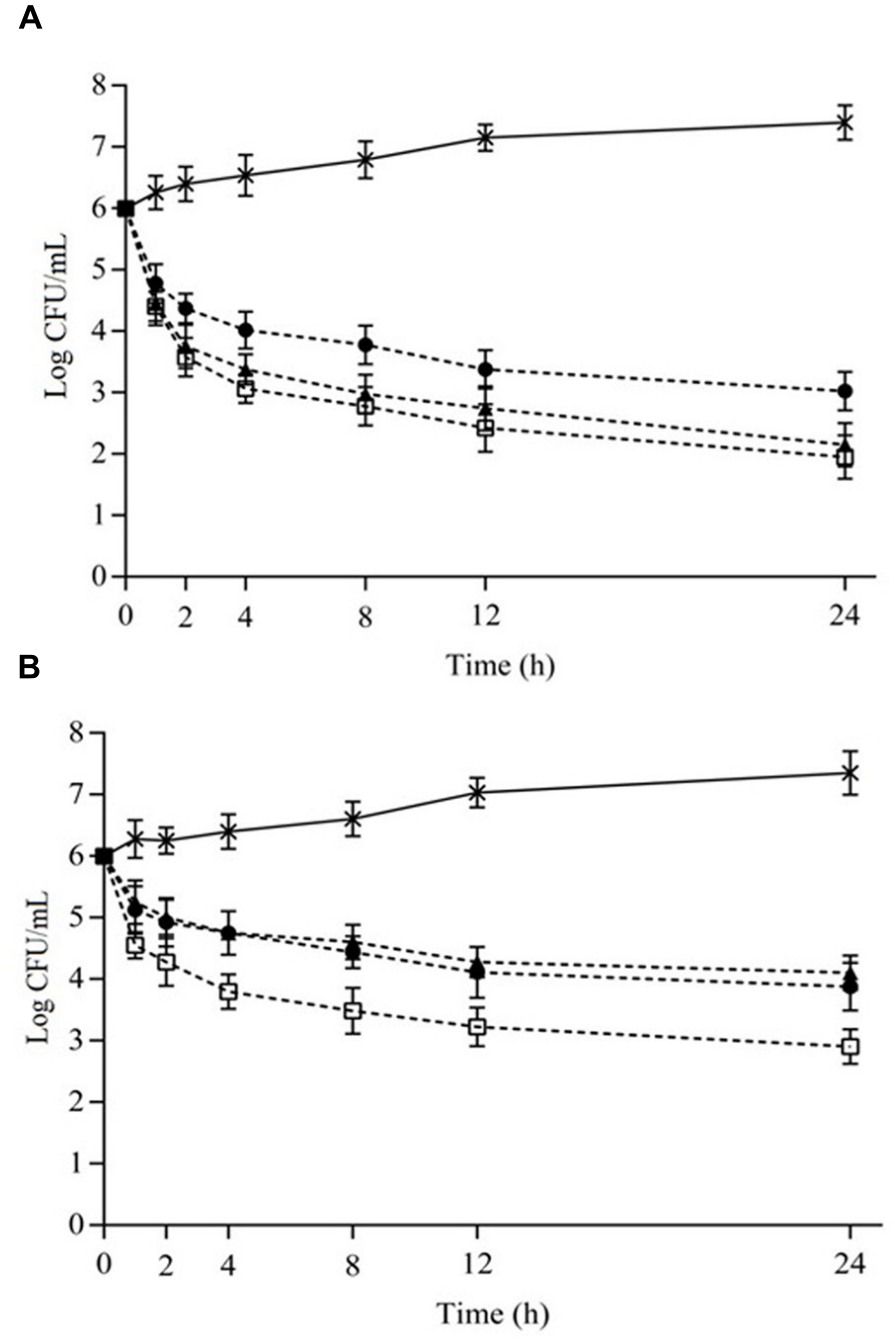
**Viable cell counts (Log CFU/mL) of *S. aureus* cocktail in meat broth (7°C) as a function of different combinations of the essential oils from *O. vulgare* L. (OVEO) and *R. officinalis* L. (ROEO) **(A)** and carvacrol (CAR) and 1,8-cineole (CIN).** **(A)**: (•) 1/8 MIC OVEO + 1/4 MIC ROEO; (□) 1/4 MIC OVEO + 1/8 MIC ROEO; (▲) 1/4 MIC OVEO + 1/4 MIC ROEO; **(B)**: (•) 1/4 MIC CAR + 1/4 MIC CIN; (□) 1/2 MIC CAR + 1/4 MIC CIN; (▲) 1/2 MIC CAR + 1/2 MIC CIN. (^∗^) Control system (without OVEO and CAR or CAR and CIN).

Viable cell counts (counts) of *S. aureus* cocktail when exposed to the different combinations of EOs or ICs in cheese and meat broths over 24 h are given in **Figures 1A,B** and **2A,B**, respectively. Systems containing the combinations of OVEO and ROEO or CAR and CIN always exhibited lower counts (*p* ≤ 0.05) compared with control systems (without EOs or ICs). All assayed combinations of essential oils and constituents caused similar decreases (*p* > 0.05) in counts of *S. aureus* cocktail over 24 h in both cheese and meat broths.

A sharp drop (*p* ≤ 0.05) in counts of *S. aureus* cocktail was observed when the combinations of OVEO and ROEO were incorporated in cheese broth. In assays with combinations of 1/8MIC and 1/4MIC, a reduction (*p* ≤ 0.05) of up to 3.7 log CFU/mL in initial *S. aureus* counts was observed after 24 h of exposure. EOs combined at these concentrations caused a higher decrease (*p* ≤ 0.05) of counts when compared to the combinations of 1/4MIC OVEO + 1/4MIC ROEO, which reduced approximately 2.9 CFU/mL the initial counts.

The incorporation of CAR and CIN in cheese broth at 1/4MIC reduced the initial counts of *S. aureus* cocktail approximately 3.0 log CFU/mL after 24 h of exposure. Lower decrease (*p* ≤ 0.05) was observed in initial counts when the cocktail was assayed in cheese broth containing combinations of 1/2MIC and 1/4MIC; for these combinations, a reduction of up to 1.8 log CFU/mL was noted after 24 h of exposure. Decreased counts in cheese broth occurred as early as 2 h of exposure to all tested combinations of essential oils or constituents, and no increase in counts was observed for the remainder of the assessed exposure time.

Similar effects were observed when *S. aureus* cocktail was cultivated in meat broth. Initial cells counts decreased (*p* ≤ 0.05) approximately 4.0 log CFU/mL after 24 h of exposure in meat broth containing OVEO and ROEO at combinations of 1/4MIC and 1/8MIC. When the EOs were incorporated in meat broth combined at 1/4 MIC, lower decrease (*p* ≤ 0.05) in counts was observed; initial counts reduced approximately 3.0 log CFU/mL after 24 h of exposure. The combination of 1/4MIC of CAR and CIN caused reduction in initial counts of *S. aureus* in meat broth of approximately 3.0 log CFU/mL after 24 h of exposure. The combinations with 1/2MIC or 1/2MIC and 1/4 MIC caused reduction of up to 2.0 log cycles in counts of *S. aureus* in meat broth. The counts decreased after 2 h of exposure to essential oils or constituents in all combinations assayed in meat broth, and no increase in counts was observed for the remainder of the assessed exposure times. *S. aureus* cocktail cultivated in cheese and meat broths without EOs or ICs showed an increase in initial *S. aureus* counts over the assessed time interval (1.0–1.5 log cycles after 24 h).

Considering these results, the combinations of OVEO and ROEO at 1/8 MIC and 1/4 MIC, respectively, and both CAR and CIN at 1/4 MIC presented bactericidal effects (≥3 log CFU/mL reduction in the initial counts, i.e., ≥99.9 killed (LaPlante, 2007) against the *S. aureus* cocktail after 24 h of exposure in either cheese or meat broth. Because of these effects, the referred combinations were tested in assays using cheese and meat samples.

### Effects on Bacterial Counts in Cheese and Meat

**FIGURE 3:**
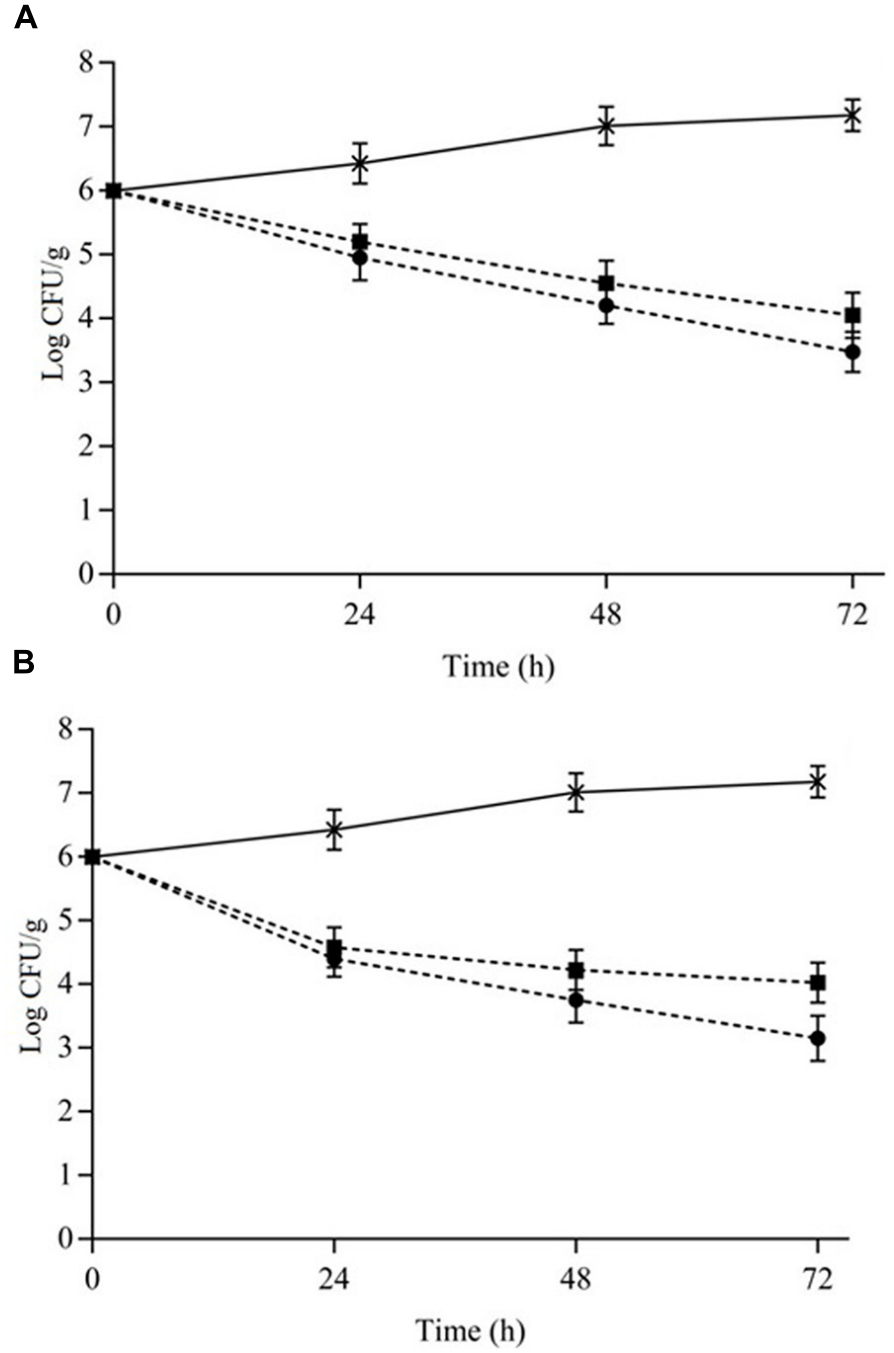
**Viable cells counts (Log CFU/mL) of *S. aureus* cocktail in cheese **(A)** and meat **(B)** at 7°C as a function of different combinations of the essential oils from *O. vulgare* L. (OVEO) and *R. officinalis* L. (ROEO) and carvacrol (CAR) and 1,8-cineole (CIN).** **(A,B)**: (•) 1/8 MIC OVEO + 1/4 MIC ROEO; (■) 1/4 MIC CAR + 1/4 MIC CIN; (^∗^) Control system (without OVEO and CAR or CAR and CIN).

The counts of *S. aureus* cocktail in cheese and meat over 72 h (at 7°C) when challenged with the combination of 1/8MIC OVEO + 1/4MIC ROEO and 1/4MIC CAR+ 1/4MIC CIN are given in **Figure 3**. After a 48 h-exposure, combined essential oils or constituents reduced the initial counts of *S. aureus* ≥2.5 log cycles in cheese (**Figure 3A**) and meat (**Figure 3B**) samples.

Similarly to observed in assays with broth, cheese and meat samples containing EOs or ICs exhibited lower *S. aureus* counts (*p* ≤ 0.05) compared to the control systems (without EOs or ICs). However, the decrease in initial counts of *S. aureus* was lower (*p* ≤ 0.05; 1–1.3 log cycles lower) in both cheese and meat samples compared to those observed in cheese and meat broth, respectively. Cheese and meat samples containing the combinations of essential oils showed a higher reduction (*p* ≤ 0.05) of counts over time (72 h) compared to reduction found in systems containing the constituents.

The essential oils from *O. vulgare* L. and ROEO in combination caused a decrease in the initial counts of *S. aureus* cocktail of approximately 1.0 CFU/g after 24 h in cheese. After 72 h of exposure this reduction increased to approximately 2.5 log CFU/g. When CAR and CIN in combination were assayed in cheese, initial counts decreased 2.0 log CFU/g after 72 h.

Reductions of up to 2.8 log CFU/g in the initial counts (*p* ≤ 0.05) of *S. aureus* cocktail was observed in meat samples containing OVEO and ROEO at the tested subinhibitory concentrations over the assessed time. Similar to assays in cheese, incorporation of combined CAR and CIN in meat caused a lower (*p* ≤ 0.05) decrease in counts of *S. aureus* cocktail in comparison to that observed in meat samples containing the same EOs combination. Initial counts decreased approximately 2.0 log cycles after 72 h of exposure when CAR and CIN were incorporated in meat samples. The reduction in bacterial counts occurred after 24 h of exposure to the combined essential oils or constituents, and no increase in counts was observed at the later assessed times for both cheese and meat samples.

### SEM Analysis

**FIGURE 4:**
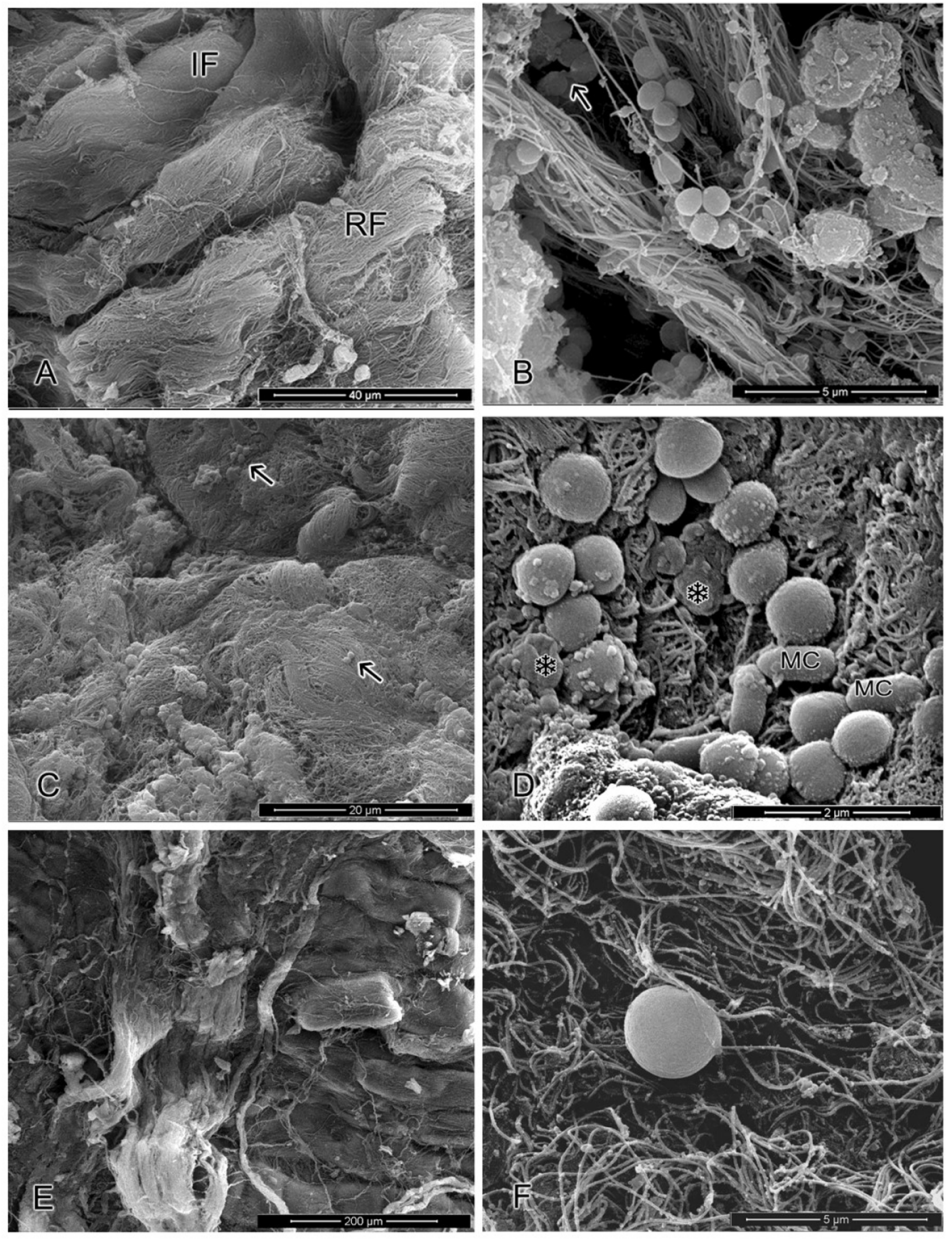
**Ultrastructural images of *S. aureus* cells cultivated in meat in meat under refrigerate temperature.** **(A,B)** Cells cultivated in meat without incorporation of essential oils from *O. vulgare* L. (OVEO) and *R. officinalis* L. (ROEO) or carvacrol (CAR) and 1,8-cineole (CIN) (control). **(A)** Low magnification showing areas on the meat surface presenting intact fibers (IF) and Rupture fibers (RF). **(B)** *S. aureus* attached to meat fibers forming grape-clusters. **(C,D)** Cells cultivated in meat containing combined OVEO and ROEO at subinhibitory concentrations (1/8MIC OVEO + 1/4MIC ROEO). **(C)** Low magnification view of meat matrix showing scarce groups of bacteria distributed along the meat surface (arrow). **(D)** High magnification image showing the presence of altered *S. aureus* and other meat contaminant bacteria (MC), as well as morphological surface alteration with shrinkage of cells (^∗^). **(E,F)** Systems containing combined CAR and CIN at subinhibitory concentrations (1/4MIC CAR + 1/4MIC CIN). **(E)** General view of meat surface with no evidence of contamination. **(F)** Detail of single bacterium adhered to a meat fiber presenting increased cell volume.

The morphology ultrastructural characteristics of *S. aureus* cells cultivated in meat samples containing or not the OVEO and ROEO or CAR and CIN in combination after 24 h of refrigerate storage are given in **Figure 4**. Cells present in control meat samples displayed under division grape-like clusters appearance and typical round morphology and smooth surface. When cells were cultivated in meat sample containing the essential oils combination (**Figure 4C**), single or small groups of two- or four-cells scarcely dispersed along the surface and only few grape-like clusters were observed. Still, in this system, *S. aureus* cells and some other possible meat naturally occurring bacteria presented altered surface morphology with appearance of cell shrinkage or like-blebbing structures (**Figure 4D**).

The meat samples containing the constituents in combination presented only scarce individual bacterial cells disperse along the matrix. Bacteria cells, when present, showed increase of their volume (**Figures 4E,F**). No noticeable morphological difference in microstructure of meat matrix was observed in OEs or ICs treated-samples as compared with non-treated meat.

## Discussion

The information regarding the inhibitory effects of essential oils or constituents combined at different subinhibitory concentrations on survival of *S. aureus* cocktail in food based-media and in food samples is relevant to understand the behavior of this pathogen when challenged in food products. In last years a number of studies focusing on antimicrobial activity of these compounds have been published (Iten et al., 2009; Azerêdo et al., 2012; Oliveira et al., 2015; Sousa et al., 2015). However, these studies frequently describe the behavior or response of bacteria in pure culture cultivated in synthetic media, which may be a non-real challenge conditions imposed by food environment.

The difference in the inhibitory effects of OVEO and ROEO (considering their often-detected MIC values) against different microorganisms has been related to their particular majority constituents profile (Burt, 2004; Barbosa et al., 2015). Given this information, the stronger inhibitory effects of OVEO against the *S. aureus* cocktail compared to those observed for ROEO, as observed in this study, could be mainly attributed to the stronger antimicrobial activity presented by its majority compound (Azerêdo et al., 2011). Although the monoterpene CAR presents some similarity with the structure of CIN because both are oxygenate monoterpenes, the higher antimicrobial activity of CAR compared to CIN, as observed here considering the detected MIC values, is already reported in previous studies (Sousa et al., 2012; Oliveira et al., 2015). In contrast with the well-described action mechanisms of CAR, the action mechanism of CIN is not yet fully clarified (Pesavento et al., 2015). CAR can uptake across the bacterial membrane, mostly due to the *ortho*-position of its hydroxyl group, blocking microbial enzymes, and interfering in systems required for the cell survival (Lambert et al., 2001; Burt, 2004). CIN has been reported to act in cell membranes, mainly due its hydrophobicity (Oliveira et al., 2015)

The essential oils from *O. vulgare* L. and ROEO, as well as CAR and CIN presented synergism interactions against the *S. aureus* cocktail in the checkerboard assay. The inhibitory effects of these synergistic interactions were also observed in assays with broths and food samples. The synergism observed between the tested antimicrobial compounds is probably a consequence of interaction mechanisms produced in sequential inhibition of a common biochemical pathway (as inhibition of protective enzymes), as well as due the combinations of cell wall active agents which cause an increase of cell membrane permeability provides the cellular uptake of other inhibitory substances or compounds (Santiesteban-López et al., 2007). Particularly, the synergism between OVEO and ROEO or CAR and CIN against *S. aureus* might be the result of an initial disturbance in the bacterial membrane structures caused by mainly CIN due the high hydrophobicity, or other constituents such as camphor and α-pinene, which helps the CAR, to cross the bacterial membranes and act directly on them. These effects may cause loss of ions and reduction of the membrane potential, loss of function of the proton pumps and ATP depletion, or damage to proteins, lipids, and organelles present within the bacteria (Di Pasqua et al., 2006; Sousa et al., 2015). Then, the lower amounts of each antimicrobial compound were required for the establishment of antimicrobial effects.

Time-kill curves of combined OVEO and ROEO or CAR and CIN in broths showed high decrease (>90–≥99.99%) in counts of S. *aureus* over time assessed. For the time-kill method, synergism is defined as a 100-fold decrease (2 log or 99% decrease) in viable cell counts after 24 h of exposure to subinhibitory concentrations of the tested antimicrobials (Iten et al., 2009). From this, all assayed combinations of OVEO and ROEO and the combination of CAR and CIN at 1/4MIC confirmed the synergistic interaction indicated by the FICI ≤ 0.5 in kill-time assays using meat and cheese broths. Curiously, for both, essential oils and constituents, the highest reductions (≥3 log cycles) were always observed in combinations of lower subinhibitory concentrations tested (1/4MIC or 1/8MIC). These results could be result of the saturation of cellular targets available that limits the action of the tested compounds in bacterial cells, after the initial membrane perturbation (Juven et al., 1994). Consequently, increased concentrations of essential oils or constituents in growth media did not represent a progressive increase in their inhibitory effects over time. It is noteworthy because the use of lower amounts in combination may be a reasonable choice to control *S. aureus* when considered the lower costs and the minimization of possible impacts on sensory aspects of foods. Still, these findings are reinforced because the available evidences that the exposure of *S. aureus* to subinhibitory concentrations of OVEO, ROEO, CAR, or CIN do not induce the increase of direct-tolerance and cross-tolerance (Gomes-Neto et al., 2012; Silva da Luz et al., 2013).

The inhibitory effects of combined essential oils or constituents at subinhibitory concentrations against the *S. aureus* cocktail in cheese and meat samples were lower than those observed in cheese and meat broth for the same combinations. Moreover, considering the decrease near to 99% in initial counts after 72 h-exposure during refrigerate storage, the assayed combinations in cheese and meat could be fully considered for control the growth of *S. aureus* because this bacterium rarely exceeds 4 log CFU/g in these products (Sergelidis et al., 2012). In cheese and meat samples the 100-fold decrease in viable count (reductions in initial counts ≥ 2 log) that define the occurrence of synergism was observed after a longer exposure-time (72 h) to tested combination of essential oils or constituents compared to that observed in broths (24 h). These lower inhibitory effects in cheese and meat compared to broths were probably related to the greater availability of nutrients in food matrices. When bacteria are challenge in an environment with greater availability of nutrients, such as cheese and meat, cell injury could be repaired faster (Gill et al., 2002; Burt, 2004). Otherwise, it is known that proteins and fatty acids can react and bind with constituents of essential oils becoming them unavailable to act in target-cells (Burt, 2004; Gutierrez et al., 2008). The physical structure of food matrices may also influenced on the dispersion of the tested compounds limiting their contact with the cells and consequently, affecting the antibacterial activity (Gutierrez et al., 2009).

The ultrastructural changes observed in *S. aureus* cells cultivated in meat samples containing the OVEO and ROEO or CAR and CIN in combination could be a result of the interaction of essential oils or constituents with components of the bacterial outer membrane causing disturbance of its structure and function. Previous studies reported that bacterial cells challenged with CAR and CIN in combination presented increased permeability of cytoplasmic membranes, roughness in their surface and appearance of structures similar to blebs (vesicles) where the cytoplasmic constituents were leakage (Oliveira et al., 2015; Sousa et al., 2015). Thus, the observed shrinkage of cells in the present study could be a result of the leakage of cytoplasmic content caused by the action of CAR and CIN in cytoplasmic membranes.

## Conclusion

The results obtained in this study demonstrated the efficacy of combined OVEO and ROEO, as well as CAR and CIN, at subinhibitory concentrations in inhibiting the survival of a *S. aureus* cocktail with the occurrence of a synergistic interaction. Application of these compounds at synergistic combinations decreased the counts of *S. aureus* in cheese and meat broth, as well as in cheese and meat samples stored at refrigerate temperature. Combined application of OVEO and ROEO or CAR and CIN induced morphological alterations on cell surface of bacteria cultivated in meat. These findings reinforce the possible exploitation of OVEO and ROEO or CAR and CIN combined at subinhibitory concentrations to control *S. aureus* in food products stored at low temperatures, particularly in cheese and meat.

## Conflict of Interest Statement

The authors declare that the research was conducted in the absence of any commercial or financial relationships that could be construed as a potential conflict of interest.
